# Ferroxitosis: A cell death from modulation of oxidative phosphorylation and PKM2-dependent glycolysis in melanoma

**DOI:** 10.18632/oncotarget.3031

**Published:** 2014-12-26

**Authors:** Alexander J. Lakhter, James Hamilton, Pierre C. Dagher, Suresh Mukkamala, Takashi Hato, X. Charlie Dong, Lindsey D. Mayo, Robert A. Harris, Anantha Shekhar, Mircea Ivan, Nickolay Brustovetsky, Samisubbu R. Naidu

**Affiliations:** ^1^ Department of Dermatology, Indiana University School of Medicine, Indianapolis, Indiana, USA; ^2^ Department of Pharmacology and Toxicology, Indiana University School of Medicine, Indianapolis, Indiana, USA; ^3^ Department of Medicine, Indiana University School of Medicine, Indianapolis, Indiana, USA; ^4^ Department of Biochemistry and Molecular Biology, Indiana University School of Medicine, Indianapolis, Indiana, USA; ^5^ Department of Pediatrics, Indiana University School of Medicine, Indianapolis, Indiana, USA; ^6^ Department of Psychiatry, Indiana University School of Medicine, Indianapolis, Indiana, USA

**Keywords:** Ferroxitosis, PKM2, HIF-1α, Warburg effect, melanoma, hypoxia and mitochondria

## Abstract

Reliance on glycolysis is a characteristic of malignancy, yet the development of resistance to BRAF inhibitors in melanoma is associated with gain of mitochondrial function. Concurrent attenuation of oxidative phosphorylation and HIF-1α/PKM2-dependent glycolysis promotes a non-apoptotic, iron- and oxygen-dependent cell death that we term ferroxitosis. The redox cycling agent menadione causes a robust increase in oxygen consumption, accompanied by significant loss of intracellular ATP and rapid cell death. Conversely, either hypoxic adaptation or iron chelation prevents menadione-induced ferroxitosis. Ectopic expression of K213Q HIF-1α mutant blunts the effects of menadione. However, knockdown of HIF-1α or PKM2 restores menadione-induced cytotoxicity in hypoxia. Similarly, exposure of melanoma cells to shikonin, a menadione analog and a potential PKM2 inhibitor, is sufficient to induce ferroxitosis under hypoxic conditions. Collectively, our findings reveal that ferroxitosis curtails metabolic plasticity in melanoma.

## INTRODUCTION

Malignant cells exploit the normal adaptive mechanisms to survive and proliferate in limited oxygen environment found within the tumor parenchyma. Hypoxia-inducible factor 1α (HIF-1α) is a key transcription factor that facilitates adaptation to hypoxic conditions [1]. The levels of HIF-1α protein are tightly controlled by the availability of oxygen and iron, which activate prolylhydroxylases-mediated hydroxylation of HIF-1α and subsequent proteasomal degradation [2]. Hypoxic conditions or limited availability of iron enable stabilization of HIF-1α protein and consecutive transcriptional activation of genes responsible for glucose uptake and glycolysis [3, 4]. While increasing the glycolytic capacity of cells, HIF-1α also limits the functions of mitochondria by diminishing the supply of acetyl-coA and NADH [5]. Although HIF-1α is a critical transcription factor that regulates glycolysis and oxidative phosphorylation to promote cell survival, a cell death mechanism that inactivates this transcription factor remains unknown.

Pyruvate kinase M2 (PKM2), an embryonic isoform predominantly expressed in malignant cells catalyzes the conversion of phosphoenolpyruvate to pyruvate. PKM2 is a key determinant of aerobic glycolysis, also known as the Warburg effect, which is commonly observed in tumor cells [6]. Although initially it was thought that PKM2 is a cytoplasmic glycolytic enzyme, subsequent studies uncovered its non-canonical nuclear functions [7-9]. ERK1/2-phosphorylated PKM2 translocates into the nucleus and promotes c-myc transcription in mediating the Warburg effect and tumorigenesis [10]. These findings underscore the critical role for both nuclear and cytoplasmic functions of PKM2 in tumor progression.

Melanomas invariably harbor mutations that constitutively activate the RAF-MEK-ERK pathway, leading to aggressive progression of the disease [11-13]. Disruption of the RAF-MEK-ERK signaling with BRAF or MEK inhibitors has shown significant clinical responses in the treatment of melanoma, yet the rapid development of resistance to these inhibitors presents a formidable challenge [14]. Although aerobic glycolysis supports tumor cell proliferation, increased mitochondrial mass and capacity enables melanoma cells to overcome the RAF-MEK-ERK pathway blockade strategy [15-18]. Therefore, targeting metabolic reprogramming may provide effective clinical strategy. Here we report a novel iron- and oxygen-dependent mechanism of cell death that is coupled to oxidative phosphorylation and HIF-1α/PKM2-dependent glycolysis in melanoma.

## RESULTS AND DISCUSSION

**Figure 1:**
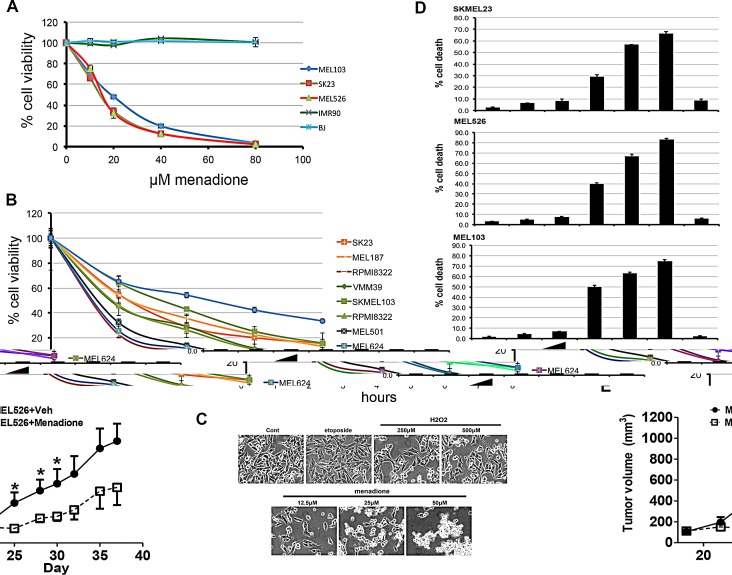
Menadione causes rapid cell death in melanoma cells A) Normal human cells, including fetal lung (IMR90) and skin (BJ) fibroblast cells as well as melanoma cell lines SK23 (wild-type BRAF/NRAS), SKMEL103 (NRAS^Q61L^) and MEL526 (BRAF^V600E^) were treated with 0-80μM menadione, and cell viability determined. Graphs show average values ± s.d. of technical triplicates from a representative experiment. B) Indicated melanoma cell lines were treated with 20μM menadione and cell viability assessed at different time points. C) Bright-field microscopy images of melanoma cells treated with H_2_O_2_, menadione (MEN), and etoposide. D) Cells treated as in c and cytotoxicity was measured by trypan blue exclusion staining (average of three independent experiments). E) MEL526 cells implanted NSG mice were treated with vehicle or menadione. Tumor volume measurements are shown. Plotted mean and SEM (n=4), (* < 0.05).

Menadione, a vitamin K metabolite [19], is detectable in human and rodent tissues, although the physiological role remains unclear. We undertook a study to evaluate the cytotoxic effects of menadione on human melanoma cells harboring mutations in BRAF (MEL526), NRAS (MEL103) and wild-type BRAF/NRAS (SKMEL23), or normal human fetal lung (IMR90) and skin (BJ) fibroblasts. Menadione significantly reduced the viability of melanoma cells in a concentration-dependent manner with an EC50 of 20μM, whereas the viability of normal cells was unchanged (Figure 1A). Subsequently, several melanoma cell lines exposed to 20μM menadione showed a dramatic reduction in viability regardless of the RAF-MEK-ERK pathway mutations (Figure 1B). Results from dye-exclusion assays revealed that menadione, but not other pro-oxidants, promoted a robust cell death (Figure 1C,D). The *in vivo* relevance of these observations was ascertained in MEL526 cells xenografted to NSG mice where menadione significantly reduced tumor growth (Figure 1E). To test the possibility of p53 activation and involvement of autophagy, melanoma cells were treated with etoposide, H_2_O_2,_ or menadione, and the cell extracts were examined by immunoblot. Menadione neither activated the p53 pathway nor induced autophagy (Figure S1). Caspase activity was unchanged by menadione, and pre-treatment with the pan-caspase inhibitor Z-VAD-FMK did not prevent its cytotoxic effects (Figure S1). Consistent with these data, menadione did not alter the mitochondrial membrane potential (Movie S1). Inhibition of necroptosis with nectrostatin-1 also did not reduce menadione-mediated cell death, in accordance with fluorescent assays of cell membrane integrity (Figure S1). These results suggest that menadione causes a form of cell death distinct from apoptosis, autophagy and necrosis.

**Figure 2:**
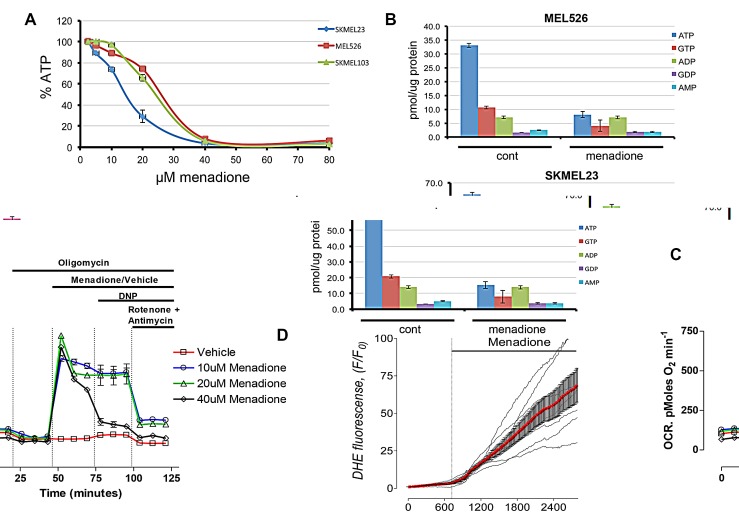
Menadione enhances oxygen consumption and depletes intracellular ATP A) Menadione promotes dose-dependent decrease in intracellular ATP levels in melanoma cells. ATP levels determined by a luminescent cell-based assay; n=3. B) HPLC determination of total nucleotides from cells that are treated with 20μM of MEN or vehicle for 1.5 hours. C) Oxygen consumption rate (OCR) was measured on a Seahorse analyzer. Oligomycin (1μM), vehicle (ethanol) or menadione (10-40μM), 2,4-dinitrophenol (DNP, 60μM), and a combination of rotenone (1μM) and antimycin A (1μM) were applied to Mel526 cells as indicated. Each data point represents mean OCR ± s.e. from 5 replicates. D) Superoxide levels measurements by DHE fluorescence in the presence of menadione.

To determine whether menadione-mediated cell death is linked to energetic catastrophe we used an ATP-coupled luminescence assay. Menadione exposure caused a dose-dependent depletion of ATP, with a nadir at 40μM (Figure 2A). These results were substantiated by HPLC-based biochemical analysis of total nucleotide from menadione-treated samples, which confirmed a dramatic reduction in ATP and GTP, with no change in the levels of other nucleotides (Figure 2B). Measurements of oxygen consumption rate (OCR) demonstrated that menadione caused a robust increase in OCR, far exceeding that of the uncoupling agent 2,4-dinitrophenol (Figure 2C). Furthermore, dihydroethidium (DHE) fluorescence assay verified menadione-induced production of superoxide (Figure 2D). Consistent with this observation, pretreatment of cells with anti-oxidants prevented the effects of menadione (Figure S2). These results suggest that menadione uncouples oxidative phosphorylation in promoting rapid cell death.

**Figure 3:**
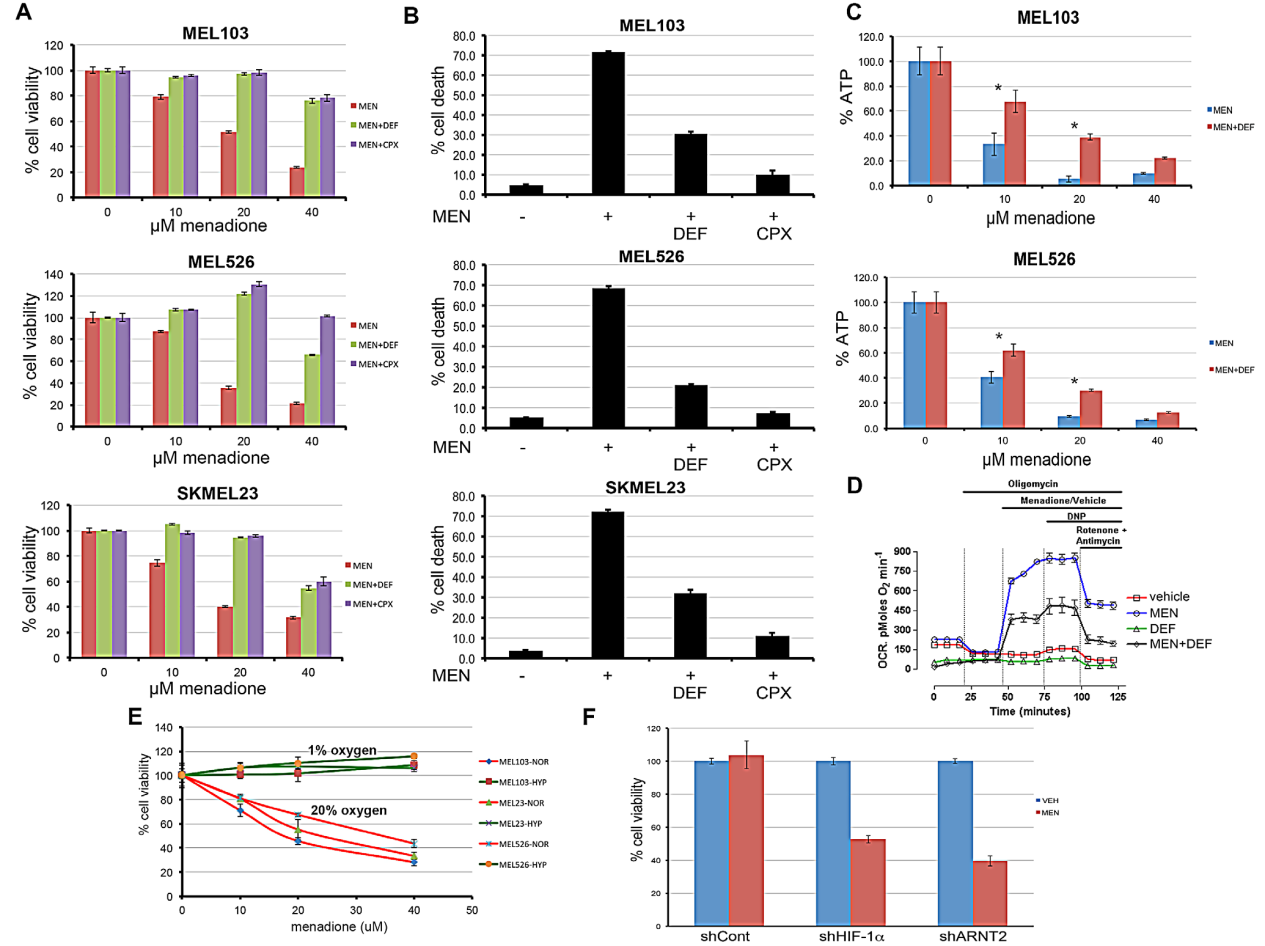
Iron chelation or hypoxic adaptation prevents the effects of menadione A) Iron chelators deferoxamine (DEF) (100μM) and ciclopirox (CPX) (10μM) prevent menadione-induced cytotoxicity as determined by resazurin-based assays. B) Prevention of menadione-induced melanoma cell death by iron chelation was assessed by trypan blue exclusion assays. C) Iron chelation partially reverses ATP depletion caused by menadione. Asterisk denotes significant difference between MEN and MEN+DEF treated samples of the corresponding MEN concentration group. *P<0.01. The error bars denote s.d. (n=5). D) OCR measurements of cells exposed to vehicle (red), 20μM menadione, (blue), 100μM deferoxamine (DEF) (green), or DEF+menadione (black). Each data point represents mean OCR ± s.e. from 5 replicates. E) Hypoxic conditions (green) prevented the effects of menadione as opposed to normoxia (red). Indicated melanoma cell lines were exposed to different doses of menadione in 1% oxygen (green) or 20% oxygen (red) and cell viability quantified. Data shown as mean ±s.d. of technical triplicates from a representative experiment. F) Melanoma cells expressing control, shHIF-1α, or shARNT2 were treated with vehicle (blue) or MEN (red) in hypoxia. HIF-1α or ARNT2 depletion sensitized cells to MEN-mediated cytotoxicity under hypoxic conditions.

Considering the critical role of mitochondria in regulation of intracellular iron, we hypothesized that menadione-induced cell death may involve iron. Perls' DAB stain [20] of menadione-treated cells indicated release of free iron (Figure S3). To test if iron chelation would block menadione-mediated cytotoxicity, cells were treated with menadione in the presence or absence of structurally unrelated iron chelators deferoxamine and ciclopirox olamine, and cell viability was determined. Iron chelation protected the cells from menadione (Figure 3A), an effect corroborated in dye-exclusion assays (Figure 3B). In addition, deferoxamine partially rescued menadione-induced loss of ATP (Figure 3C) and significantly blunted menadione-mediated increase in OCR (Figure 3D). Although menadione was cytotoxic to lung (H1299) and cervical cancer (C33a) cell lines, deferoxamine did not confer protection, suggesting that iron chelation is not sufficient to overcome the effects of menadione in these non-melanoma cell lines. Moreover, these results support the interpretation that the effects observed in melanoma cells are biological and not due to drug interactions (Figure S4). To test the involvement of known iron regulators, melanoma cells were depleted of ACO1, ACO2, ACO3, FTMT, FXN and MFI2, and cell viability in presence of menadione was determined (Figure S5). Depletion of these iron regulators did not significantly change the outcome of menadione-induced cytotoxicity. We propose that the mechanism of ferroxitosis is distinct from that of ferroptosis [21], as the latter does not produce mitochondrial ROS and there is no change in the levels of ATP. Collectively, these results suggest that menadione targets mitochondria to cause an iron- and oxygen-driven cytotoxic process that we term ferroxitosis.

Iron chelators are commonly used as hypoxia mimetics [3]. This led to the consideration that hypoxia may block menadione-induced ferroxitosis. Menadione decreased cell viability in normoxia, yet this effect was completely blocked by hypoxia (1% O_2_), suggesting that reliance on maximal mitochondrial respiration is essential for menadione-induced cell death (Figure 3E). These results are consistent with the reported observations that the hypoxic transcription program limits acetyl-CoA availability for the TCA cycle and reduces the efficiency of electron transfer, thereby collectively minimizing mitochondrial function in hypoxic conditions [5, 22, 23]. To test whether hypoxia transcription factor HIF-1α is responsible for this protective effect, MEL526 cells were transduced with lentivirus encoding control, HIF-1α, or HIF-1α transcriptional subunit ARNT2 shRNAs, and exposed to menadione. Depletion of either HIF-1α or ARNT2 restored menadione sensitivity in hypoxia (Figure 3F). These observations are in agreement with studies showing that HIF-1α-null mouse embryonic fibroblasts show elevated oxygen consumption and ATP production in hypoxia, revealing a reverse metabolic switch from glycolysis to oxidative phosphorylation [22, 24]. We conclude that activation of HIF-1α protects cells from menadione-induced ferroxitosis by minimizing oxidative phosphorylation, while attenuation of the HIF-1α pathway forces cells to use mitochondria for energy production, thereby restoring sensitivity to menadione.

**Figure 4:**
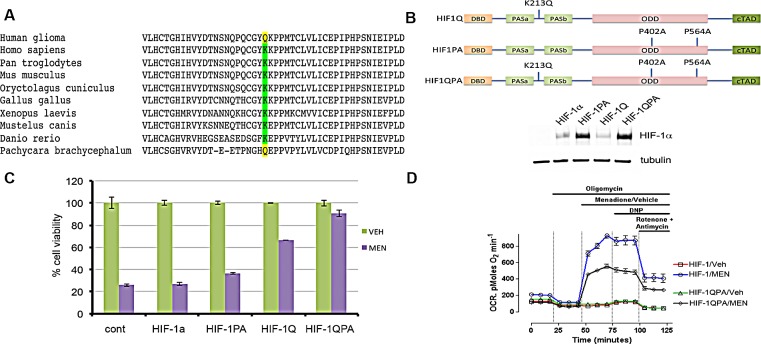
K213Q HIF-1α mutant attenuates the cytotoxic effects of menadione A) Sequence alignment of HIF-1α protein showing conservation of K213. B) Schematic representation of HIF-1α, K213Q, P402A/P564A, or a combination of these mutations. Immunoblot showing relative levels of HIF-1α protein expression in melanoma cells transduced with indicated HIF-1α viral vectors. C) Overexpression of HIF-1QPA mutant but not the individual mutants enabled melanoma cells to resist MEN-induced cytotoxicity. Data shown as mean ± s.d. of technical triplicates from a representative experiment. D) OCR measurements show that HIF-1QPA expressing cells blunt the effects of menadione-induced increase in oxygen consumption. The error bars denote s.e.m. (n=5).

Mining of the publicly available COSMIC cancer genome catalog (http://cancer.sanger.ac.uk/cosmic/mutation/overview?id=1559432), which includes the data set from The Cancer Genome Atlas revealed a novel recurrent mutation, K213Q, in the dimerization domain of HIF-1α in human glioma samples (Table S1). While the HIF-1α oxygen sensing domain, where prolyl hydroxylases target P402 and P564 to promote degradation [25, 26], is relatively well-studied, the dimerization domain that harbors K213Q is not. We questioned whether the K213Q amino acid substitution may have an evolutionary significance. While lysine 213 of the HIF-1α protein is highly conserved from humans to zebrafish, Antarctic deep-water fish *Pachycara brachycephalum* harbors glutamine instead of lysine at 213 (Figure 4A). Metabolic adaptation enables this fish to dwell in an oxygen-limited environmental niche [27]. Although publicly available dataset has not revealed the presence of K213Q mutation in melanoma samples, studies suggest that HIF-1α-dependent glycolytic program offers metabolic plasticity and growth advantage for melanoma cells [28-33]. To test the effects of K213Q on the stability of the HIF-1α protein, MEL526 cells were lentivirally transduced to express wild type HIF-1α, the degradation-resistant P402A/P564A (HIF-1PA), K213Q (HIF-1Q), and the combined K213Q/P402A/P564A (HIF-1QPA). Immunoblot analysis showed an increase in the stability of the HIF-1PA and HIF-1QPA mutants (Figure 4B). To assess the biological effects of K213Q, melanoma cells stably expressing wild-type HIF-1α, HIF-1Q, HIF-1PA, or HIF-1QPA were exposed to menadione. Among these stable cells, an increased protection against menadione by HIF-1Q-expressing cells and a complete prevention of menadione-induced ferroxitosis by HIF-1QPA-expressing cells were recorded (Figure 4C). Therefore, we asked if HIF-1QPA would suppress the enhanced rate of oxygen consumption by menadione. We found that melanoma cells expressing HIF-1QPA reduced menadione-mediated increase in OCR by 50% compared to cells expressing wt-HIF-1α (Figure 4D). These results lead us to propose that K213Q of HIF-1α found in tumor cells or organisms living in limited oxygen enable them to reduce mitochondrial respiration in adaptation to hypoxic conditions.

**Figure 5:**
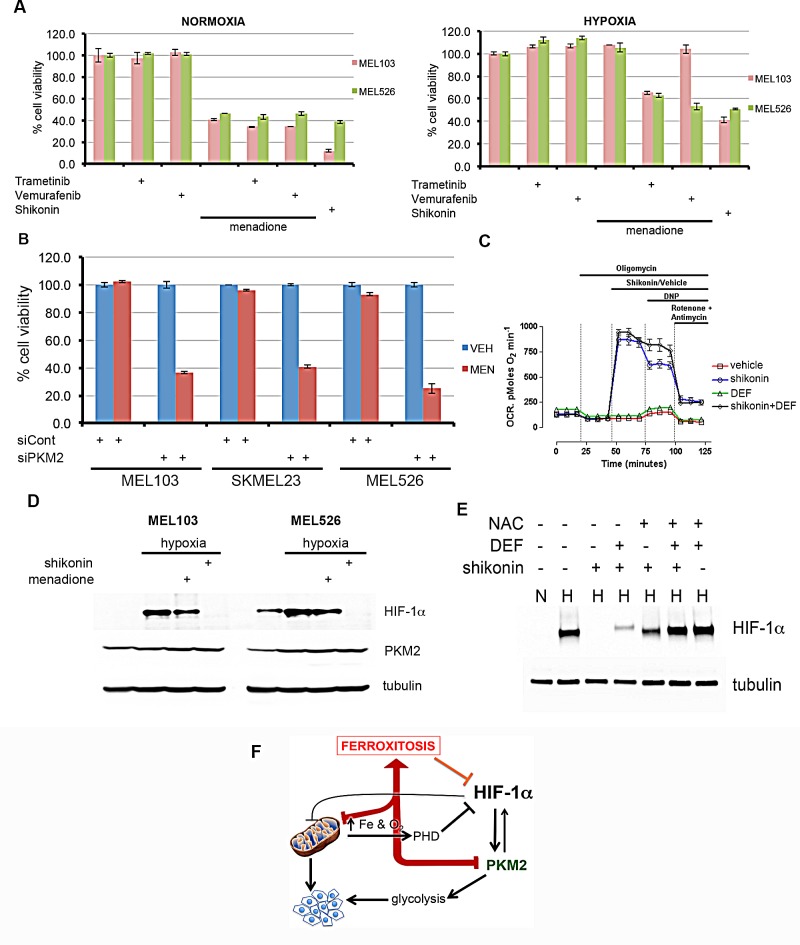
Activation of ferroxitosis involves concurrent inhibition of oxidative phosphorylation and PKM2-dependent glycolysis in melanoma A) MEL526 (BRAF^V600E^) or MEL103 (NRAS^Q61L^) cells exposed to MEK inhibitor trametinib, BRAF inhibitor vemurafenib, menadione, shikonin, or a combination of these drugs as shown in normoxia or hypoxia, and cell viability measured. B) Indicated melanoma cells were transfected with control or PKM2 siRNA; treated with vehicle (blue) or MEN (red) in hypoxic conditions and cell viability assessed. C) OCR measurements of melanoma cells exposed to vehicle (DMSO) or shikonin, shikonin+DEF. Each data point represents mean OCR ± s.e. from 5 replicates. D) Western blot analysis of melanoma cells treated with menadione or shikonin under hypoxic conditions by blotting with HIF-1α or tubulin antibodies. E) MEL103 cell extracts from normoxia (N) or hypoxia (H) exposed to shikonin, N-acetyl cysteine (NAC), deferoxamine (DEF) or in combination as shown, and blotted with HIF-1α antibody. F) Activation of ferroxitosis from concurrent inhibition of oxidative phosphorylation and PKM2-dependent glycolysis.

To further characterize the mechanism of ferroxitosis, we screened analogs of menadione with the aim of identifying a compound possessing the anti-mitochondrial activity of menadione while blunting the protective effects of iron chelation (Figure S6). One such compound, shikonin consistently reduced cell viability even in the presence of iron chelator. Shikonin has been described as a selective inhibitor of PKM2 activity and treatment of mice with shikonin reduced PKM2-dependent lactate production [34, 35]. Based on the reported observations that nuclear function of PKM2 requires ERK activity [10] and that the RAF-MEK-ERK pathway is hyperactive in melanoma [11-13], we hypothesized that BRAF and MEK inhibitors may sensitize melanoma cells to menadione in hypoxic conditions. Cell viability assays from several combinations of these drugs in normoxia and hypoxia revealed that MEK inhibitor (trametinib) or BRAF inhibitor (vemurafenib) by themselves were not cytotoxic (Figure 5A). However, when combined with these inhibitors, menadione significantly decreased cell viability in hypoxia. BRAF inhibitor was selective in sensitizing mutant BRAF (MEL526) but not mutant NRAS (MEL103) melanoma cells to menadione. Because menadione-mediated ferroxitosis requires maximal mitochondrial respiration, it is likely that trametinib and vemurafenib inhibit ERK activity and disable the nuclear function of PKM2 in sensitizing cells to menadione. In agreement with these observations, menadione analog shikonin, a known inhibitor of PKM2 [34, 35], was sufficient to dramatically reduce the viability of cells in hypoxia. If a compromise in PKM2 function is essential for sensitizing cells to menadione, we reasoned that PKM2 depletion should sensitize melanoma cells to menadione in hypoxia. Recapitulating the effects of shikonin, PKM2 knockdown restored the cytotoxic effects of menadione in hypoxia (Figure 5B). These results suggest that depletion of PKM2 in hypoxia forces cells to utilize mitochondria for energy production, thus rendering these cells vulnerable to menadione-induced ferroxitosis. Consistent with these premises, oxygen consumption measurements revealed that unlike menadione, shikonin-induced increase in OCR was unaffected by iron chelation (Figure 5C). Furthermore, we found that exposure of melanoma cells to shikonin reduced the levels of lactate under hypoxic conditions (Figure S7).

Since shikonin is a bifunctional compound that inhibits PKM2 and also targets mitochondria, we asked whether ferroxitosis induced by shikonin would destabilize HIF-1α. Immunoblot assays of melanoma cells showed shikonin, but not menadione destabilize HIF-1α in hypoxia (Figure 5D). To test whether shikonin-mediated HIF-1α destabilization is due to available iron and enhanced oxygen consumption associated oxidative stress, MEL103 cells were treated with iron chelator, N-acetyl cysteine (NAC), or a combination of these agents, and the effects of shikonin on HIF-1α stability in hypoxia was assessed. Although iron chelation or NAC was not sufficient to completely negate the effects of shikonin, NAC combined with deferoxamine reversed HIF-1α destabilization (Figure 5E).

It has been recognized that mitochondria play a key role in the pathophysiology of various diseases including cancer [36, 37]. For example, gain of mitochondrial mass and capacity are thought to contribute to melanoma developing resistance to treatment with BRAF inhibitors [15-18]. These reports led to the suggestion that targeting oxidative phosphorylation with antidiabetic biguanide may delay resistance development to BRAF inhibitors [15]. Although high concentrations of biguanide phenformin seem to augment the effects of BRAF inhibitor [38], clinical benefits of these findings are not yet known. Our results demonstrate that menadione specifically targets oxidative phosphorylation, which could in turn improve the therapeutic efficacy of BRAF and/or MEK inhibitors. Recognizing the complexity of both nuclear and cytoplasmic functions of PKM2, we propose that targeting PKM2-dependent glycolysis and oxidative phosphorylation could be a novel therapeutic approach for neuroectodermal tumors. Considering that highly proliferative tumor cells rely heavily on PKM2 for the anabolic and energy requirements, specific targeting of PKM2 may have less impact on normal cells. Collectively, our findings establish that activation of ferroxitosis in the context of PKM2 inhibition will not only inactivate oxidative phosphorylation (Figure 5F), but it will also serve as feed-forward loop to destabilize HIF-1α and presents a novel strategy to combat melanoma.

## MATERIALS AND METHODS

### Cell culture

Cell lines were maintained at 37 °C in a humidified atmosphere at 5% CO2 and grown in RPMI 1640 or DMEM growth media (Invitrogen) supplemented with 10% fetal bovine serum (Sigma), 50 units ml^−1^ penicillin and 50 μg ml^−1^ streptomycin (Invitrogen). The following cell lines were maintained in RPMI 1640: SK23, MEL501, MEL526, MEL624. The following cell lines were maintained in DMEM: BJ, IMR90, MEL103, MEL187, RPMI 8322, VMM39, WM2664. Hypoxic conditions (1% O2) were achieved in a Ruskinn *in-vivo2 400* hypoxia chamber, by supplementing ambient air with balanced N_2_ and CO_2_.

### Animal studies

NSG (NOD/scid/IL2Rgnull) mice were bred at IU Simon Cancer Center In-Vivo Therapeutic Core facility. 8 week old male animals were subcutaneously implanted with 1 million of MEL526 cells in 100μl serum free media into the right hind flank. Tumors were allowed to develop for 21 days after which the mice were randomized into the control and treatment groups (n=4). Treatments of vehicle (DMSO) or MEN (15mg/kg body weight) were administered via intraperitoneal injection three times a week [39]. Xenograft size was measured three times a week with a digital caliper and the ellipsoidal tumor volumes were recorded. All procedures were conducted in accordance with the principles outlined in the NIH Guide for the Care and Use of Laboratory Animals and were approved by the Indiana University Institutional Animal Care and Use Committee (IACUC).

### Cell based assays of viability and ATP concentration

Cells were seeded at density of 1 × 10^4^ cells per well of a 96 well plate (Corning) in technical triplicates per condition. Viability was assayed using resazurin reagent (Biotium) in accordance with manufacturer's protocol. ATP and caspase activity were measured using CellTiter-Glo reagent (Promega). Fluorescent and luminescent signals were read on Synergy H1 microplate reader (BioTek Instruments).

### Cellular Respirometry

Oxygen consumption rate (OCR) of cultured cells was measured with an XF24 extracellular flux analyzer (Seahorse Bioscience). Cells were seeded at 4 × 10^4^ cells per well one day before the experiment and all experiments were performed at 37°C in a bath solution consisting of RPMI:DMEM (1:2) supplemented with 3% FBS. Following three baseline OCR measurements, wells were sequentially injected with oligomycin, menadione or vehicle, 2,4-dinitrophenol, and rotenone + antimycin A. Once injected, each compound was present in the bath medium for the duration of the experiment. Three OCR measurements were performed after each injection. To ensure that the culture maintained sufficient oxygenation, a 3-minute mix, 2-minute wait cycle occurred prior to each 3-minute measurement.

### Immunoblot analysis

Whole-cell extracts were prepared in urea buffer (6 M urea, 100 mM sodium dihydrophosphate, 10 mM Tris pH 8). SDS-PAGE was performed using TGX gradient gels (Bio-Rad) and transferred onto PVDF (Millipore) using TransBlot SD semi-dry transfer apparatus (Bio-Rad) as per manufacturer's guidelines. The blots were probed with following antibodies: p21, p53, and PUMA (Santa Cruz), HIF-1a (R&D Systems), LC3 (Novus), PKM2 (Cell Signaling), p62 and tubulin (Sigma). Blot images were captured on ImageQuant LAS 4000 digital imaging system (GE Healthcare, Piscataway, NJ).

### HPLC analysis of total nucleotides

Following treatment, medium was aspirated and the cells washed three times with ice-cold PBS. Extraction was done by scraping the cells in 180 μl ice-cold acetonitrile followed by 420 μl cold water. The soluble and precipitated fractions were centrifuged at 16000 g for 10 min at –20^o^ C. The supernatant fraction, kept on ice, was then gassed with N2 for 30 min to evaporate acetonitrile. The pellet was solubilized with 1 N NaOH and the protein content analyzed with Coomassie Blue assay (Pierce Chemical, Rockford, IL). The column used was a 4 μm Nova-Pack C18 cartridge (100 mm by 8 mm ID), equipped with a radial compression chamber (Waters, Millford, MA). The buffer consisted of 20% acetonitrile, 10 mM ammonium phosphate and 2 mM PIC-A ion pairing reagent (Waters) and was run isocratically at 2 ml/min (10). Samples were diluted in half and the injection volume was 100 μl. A HP Chemstation model 1100 was used (Hewlett-Packard, Wilmington, DE), and the UV detector set at 254 nm. HPLC grade nucleotide standards were used to calibrate the signals. They were run daily because the retention of the column varied with time. Internal standards were added to the samples to test recovery. It exceeded 90% for all nucleotides.

### siRNA knockdown

Gene knockdown was done using PepMute (SignaGen) with custom-made dsiRNA (IDT) based on published sequences. Briefly, cells were seeded into 6-well plates at a density of 3 × 10^5 cells per well. siRNA complexes were prepared at 20 nM siRNA in according to the manufacturer's instructions.

### shRNA-mediated silencing

The lentiviral shRNA expression plasmids were from Sigma. The shRNAs targeting HIF-1a is TRCN0000010819 and ARNT is TRCN0000356097. The control shRNA is the pLKO.1 - TRC control (Addgene, plasmid 10879). The production of viral particles and transduction of target cells was conducted as described at Broad Institute webpage.

### Statistical Analysis

The DHE fluorescence plots show F/F0 traces from individual cells (thin, grey traces) as well as the average F/F0 signal (thick, red trace). The average DHE F/F0 traces are mean ±SEM.

## SUPPLEMENTARY MATERIAL FIGURES TABLE AND MOVIES










