# Supplementary material for: Ferroxitosis: A cell death from modulation of oxidative phosphorylation and PKM2-dependent glycolysis in melanoma
Source: Oncotarget. 2014 Dec 26;5(24):12694–703. doi: 10.18632/oncotarget.3031 (PMC4350353; doi:10.18632/oncotarget.3031)
Supplement: Supplementary file 1 [file oncotarget-05-12694-s001.pdf]

Ferroxitosis: A cell death from modulation of oxidative phosphorylation and PKM2-dependent glycolysis in melanoma

Supplementary Material

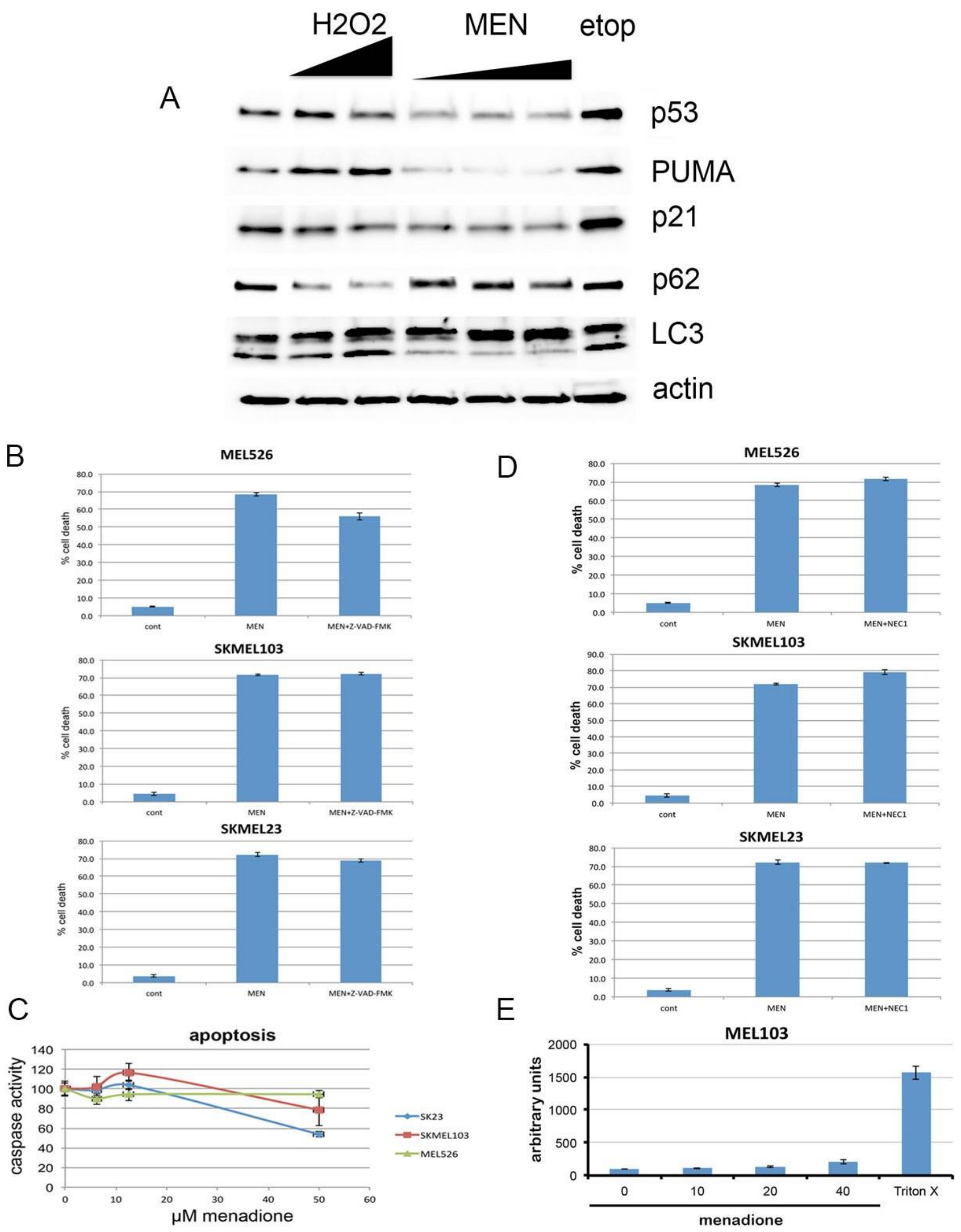

Figure S1: Menadione promotes a distinct cell death. A) MEL103 cells were treated with 250uM and 500uM H_2O_2, 12.5, 25, and 50 uM of MEN, and 40uM of etoposide. The lysates were blotted for p53, PUMA, p21, p62, LC3, and actin. **B)** MEL103, SKMEL23, and MEL526 cells were exposed to MEN (20uM) -/+ Z-VAD-FMK (100uM) for 5 hours and cell death was assessed by Trypan blue dye exclusion assay. Viable and non-viable cells were independently counted (n=4). **C)** Indicated melanoma cells were treated with increasing concentrations of MEN and caspase 3/7 activity was determined in a luminescence-coupled assay. **D)** MEL103, SKMEL23, and MEL526 cells were exposed to MEN (20uM) -/+

Necrostatin1 (25uM) for 5 hours and cell death was assessed by Trypan blue dye exclusion assay. **E)** MEL103 cells were treated with increasing concentrations of MEN and cytotoxicity was measured using cell-impermeable bis-AAF-R110 compound in CytoTox-Fluor assay. Triton X-100 (0.05%) was used as positive control.

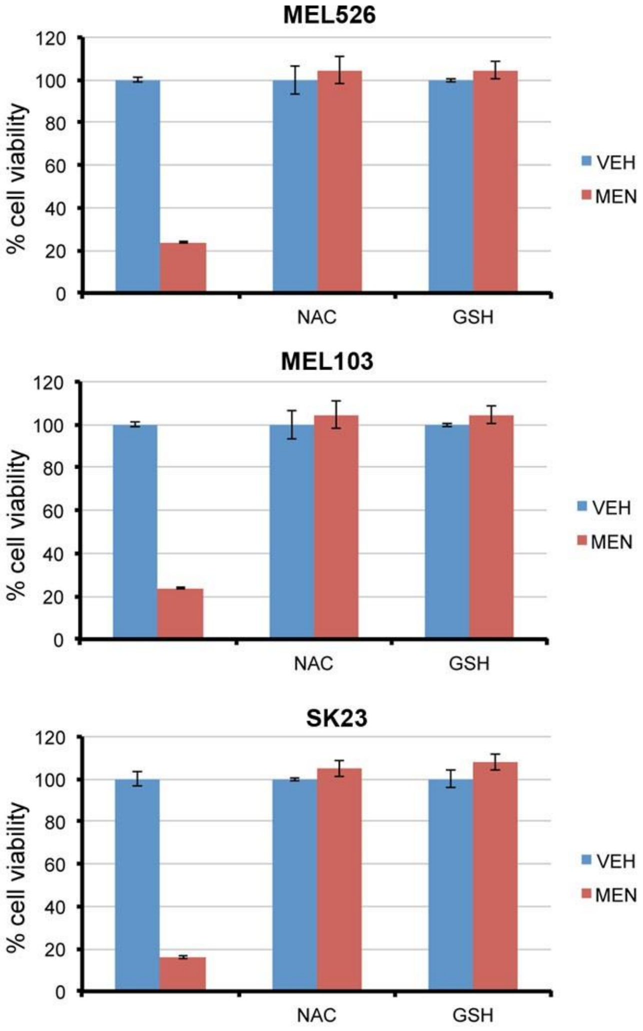

Figure S2: Antioxidants GSH and NAC prevent the effects of menadione. MEL103, SKMEL23, and MEL526 cells were treated with MEN (20uM) in absence or presence of NAC (10mM) or GSH (5mM) for 5 hours. Viability was assessed by resazurin and data plotted as mean ±s.d. of the mean in technical triplicates.

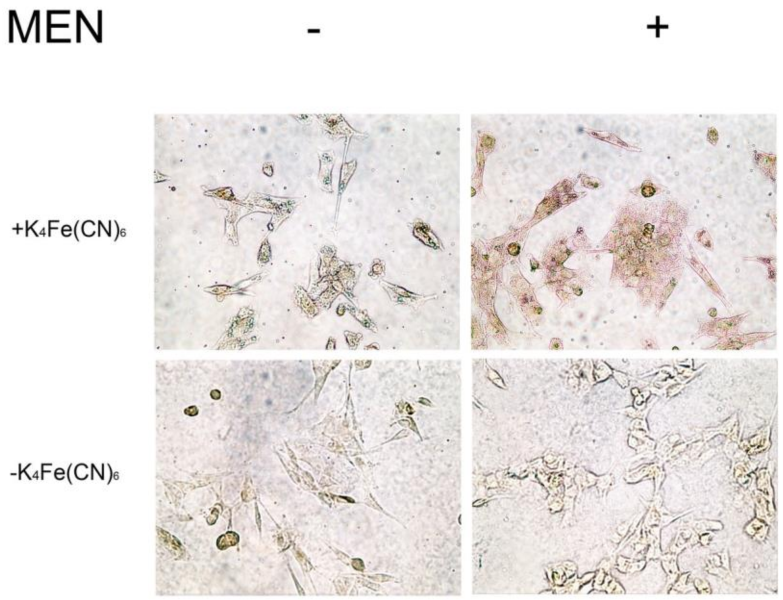

Figure S3: Menadione promotes release of intracellular iron. MEL526 cells, treated with vehicle or MEN (20uM) for 2 hours, were stained for ferric iron by Pearl's stain with DAB amplification. Control samples (lower panel) were exposed to DAB, but without Pearl's reagent. Imaging was done on Nikon Eclipse 80i microscope fitted with QImaging Retiga Exi camera.

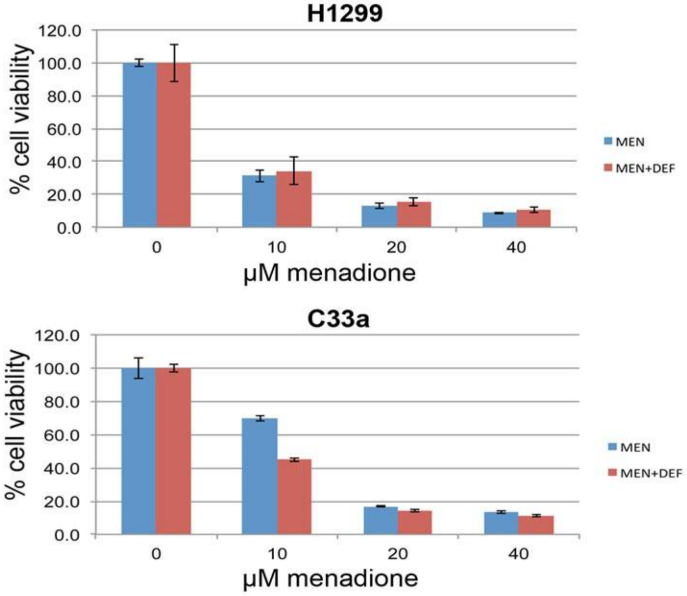

Figure S4: Iron chelation does not confer protection against menadione in non-melanoma cell lines. H1299 non-small cell lung cancer cells, and C33a cervical carcinoma cells were treated with vehicle or MEN at indicated concentrations in the absence or presence of DEF (100 uM). Viability was assessed by resazurin and data plotted as mean of technical triplicates ±s.d. in a representative experiment.

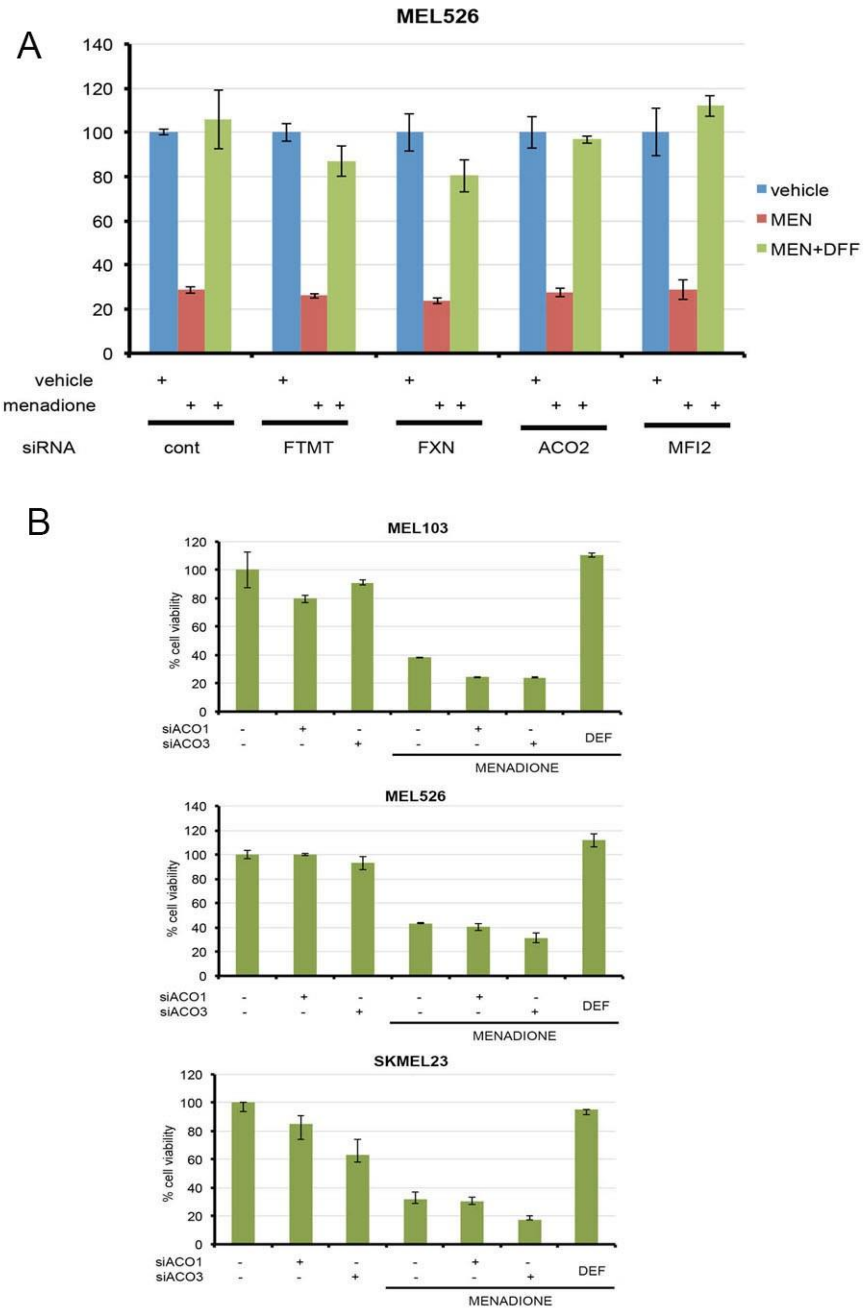

Figure S5: Depletion of iron regulatory proteins does not significantly alter the effects of menadione. A, Cell viability of MEL526 cells transfected with control siRNA or siRNAs targeting iron regulator proteins FTMT, frataxin (FXN), aconitase 2 (ACO2), and MFI2 treated with vehicle (DMSO), MEN (20uM), or MEN (20uM) and DEF (200uM). Error bars represent ±s.d.of the mean, in technical triplicates in a representative experiment. **B,** Cell viability of MEL103, SKMEL23, and MEL526 cells in context of control, aconitase 1 (ACO1), or aconitase 3 (ACO3) knockdowns. Error bars represent ±s.d. of the mean, in technical triplicates in a representative experiment.

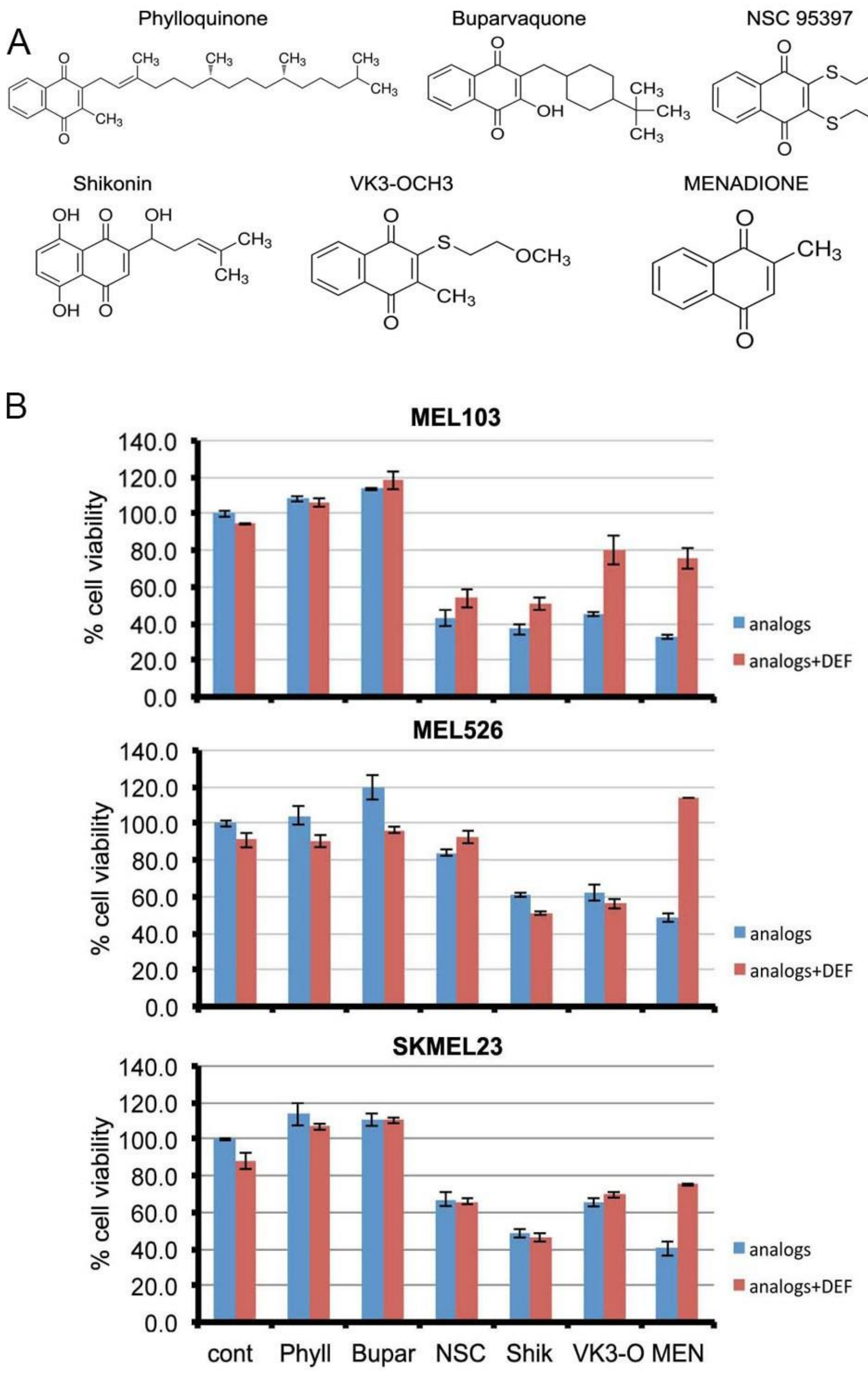

Figure S6: Shikonin decreases melanoma cell viability even in the presence of iron chelation. A, Analogues of menadione. **B,** Cell viability of MEL103, SKMEL23, and MEL526 cells treated with 25uM of each of the compounds in the absence or presence of DEF (100uM). Error bars represent ±s.d. of the mean, in technical triplicates in a representative experiment.

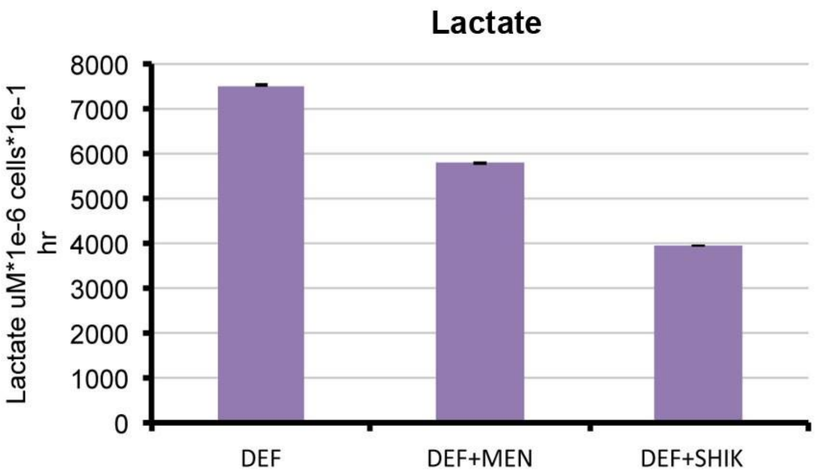

Figure S7: Shikonin reduces lactate production in melanoma cells. MEL526 cells were treated with DEF (100uM) for 2 hours, then MEN (20uM) or shikonin (25uM) were added as indicated in the figure. After 2 hours, media was collected and lactate concentration was measured using L-lactate Assay Kit. Error bars represent ±s.d. of the mean, in technical triplicates in a representative experiment.

Table S1, HIF-1a K213Q mutation in human glioma retrieved from COSMIC cancer genome catalog.

Sample Name	Gene name	Transcript	Primary Tissue	Histology	Zygosity	Sample Source
TCGA-02-0001-01	HIF1A	ENST00000337138	central nervous system	glioma	Heterozygous	Tumour Sample
TCGA-02-0003-01	HIF1A	ENST00000337138	central nervous system	glioma	Heterozygous	Tumour Sample
TCGA-02-0027-01	HIF1A	ENST00000337138	central nervous system	glioma	Heterozygous	Tumour Sample
TCGA-02-0033-01	HIF1A	ENST00000337138	central nervous system	glioma	Heterozygous	Tumour Sample
TCGA-02-0034-01	HIF1A	ENST00000337138	central nervous system	glioma	Heterozygous	Tumour Sample
TCGA-02-0047-01	HIF1A	ENST00000337138	central nervous system	glioma	Heterozygous	Tumour Sample
TCGA-02-0069-01	HIF1A	ENST00000337138	central nervous system	glioma	Heterozygous	Tumour Sample
TCGA-02-0085-01	HIF1A	ENST00000337138	central nervous system	glioma	Heterozygous	Tumour Sample
TCGA-02-0102-01	HIF1A	ENST00000337138	central nervous system	glioma	Heterozygous	Tumour Sample
TCGA-02-0113-01	HIF1A	ENST00000337138	central nervous system	glioma	Heterozygous	Tumour Sample
TCGA-06-0129-01	HIF1A	ENST00000337138	central nervous system	glioma	Heterozygous	Tumour Sample
TCGA-06-0130-01	HIF1A	ENST00000337138	central nervous system	glioma	Heterozygous	Tumour Sample
TCGA-06-0132-01	HIF1A	ENST00000337138	central nervous system	glioma	Heterozygous	Tumour Sample
TCGA-06-0133-01	HIF1A	ENST00000337138	central nervous system	glioma	Heterozygous	Tumour Sample
TCGA-06-0138-01	HIF1A	ENST00000337138	central nervous system	glioma	Heterozygous	Tumour Sample
TCGA-06-0173-01	HIF1A	ENST00000337138	central nervous system	glioma	Heterozygous	Tumour Sample
TCGA-06-0178-01	HIF1A	ENST00000337138	central nervous system	glioma	Heterozygous	Tumour Sample
TCGA-06-0185-01	HIF1A	ENST00000337138	central nervous system	glioma	Heterozygous	Tumour Sample
TCGA-06-0187-01	HIF1A	ENST00000337138	central nervous system	glioma	Heterozygous	Tumour Sample
TCGA-06-0188-01	HIF1A	ENST00000337138	central nervous system	glioma	Heterozygous	Tumour Sample
TCGA-06-0190-01	HIF1A	ENST00000337138	central nervous system	glioma	Heterozygous	Tumour Sample
TCGA-06-0195-01	HIF1A	ENST00000337138	central nervous system	glioma	Heterozygous	Tumour Sample
TCGA-06-0208-01	HIF1A	ENST00000337138	central nervous system	glioma	Homozygous	Tumour Sample
TCGA-06-0214-01	HIF1A	ENST00000337138	central nervous system	glioma	Heterozygous	Tumour Sample
TCGA-06-0237-01	HIF1A	ENST00000337138	central nervous system	glioma	Heterozygous	Tumour Sample
TCGA-08-0244-01	HIF1A	ENST00000337138	central nervous system	glioma	Heterozygous	Tumour Sample
TCGA-12-0619-01	HIF1A	ENST00000337138	central nervous system	glioma	Heterozygous	Tumour Sample

Movie S1, Menadione does not depolarize mitochondria. Mitochondrial membrane potential was assessed by live cell microscopy using TMRM. Cells were pretreated with vehicle or menadione for one hour and imaged for two hours in the presence of TMRM.

Movie S1A) MEL526 cells exposed to vehicle
Movie S1B) MEL526 cells exposed to menadione
Movie S1C) MEL103 cells exposed to vehicle
Movie S1D) MEL103 cells exposed to menadione

Experimental Procedures

Cell culture. Cell lines were maintained at 37 °C in a humidified atmosphere at 5% CO2 and grown in RPMI 1640 or DMEM growth media (Invitrogen) supplemented with 10% fetal bovine serum (Sigma), 50 units ml^{-1} penicillin and 50 µg ml^{-1} streptomycin (Invitrogen). The following cell lines were maintained in RPMI 1640: SK23, MEL501, MEL526, MEL624. The following cell lines were maintained in DMEM: BJ, IMR90, MEL103, MEL187, RPMI 8322, VMM39, WM2664. Hypoxic conditions (1% O2) were achieved in a Ruskinn *in-vivo2 400* hypoxia chamber, by supplementing ambient air with balanced N_2 and CO_2.

Animal studies. NSG (NOD/scid/IL2Rgnull) mice were bred at IU Simon Cancer Center In-Vivo Therapeutic Core facility. 8 week old male animals were subcutaneously implanted with 1 million of MEL526 cells in 100µl serum free media into the right hind flank. Tumors were allowed to develop for 21 days after which the mice were randomized into the control and treatment groups (n=4). Treatments of vehicle (DMSO) or MEN (15mg/kg body weight in DMSO) were administered via intraperitoneal injection three times a week. Xenograft size was measured three times a week with a digital caliper and the ellipsoidal tumor volumes were recorded. All procedures were conducted in accordance with the principles outlined in the NIH Guide for the Care and Use of Laboratory Animals and were approved by the Indiana University Institutional Animal Care and Use Committee (IACUC).

Cell based assays for viability, ATP concentration, caspase activity, and necrosis. Cells were seeded at density of 1 x 10^4 cells per well of a 96 well plate (Corning) in technical triplicates per condition. Viability was assayed using rezazurin reagent (Biotium) in accordance with manufacturer's protocol. ATP and caspase activity were measured using CellTiter-Glo reagent (Promega). Cellular necrosis was assessed using CytoTox-Fluor assay (Promega). Fluorescent and luminescent signals were read on Synergy H1 microplate reader (BioTek Instruments).

Cellular Respirometry. Oxygen consumption rate (OCR) of cultured cells was measured with an XF24 extracellular flux analyzer (Seahorse Bioscience). Cells were seeded at 4 x 10^4 cells per well one day before the experiment and all experiments were performed at 37°C in a bath solution consisting of RPMI:DMEM (1:2) supplemented with 3% FBS. Following three baseline OCR measurements, wells were sequentially injected with oligomycin, menadione or vehicle, 2,4-dinitrophenol, and rotenone + antimycin A. Once injected, each compound was present in the bath medium for the duration of the experiment. Three OCR measurements were performed after each injection. To ensure that the culture maintained sufficient oxygenation, a 3-minutes mix, 2-minutes wait cycle occurred prior to each measurement that lasted for 3 minutes.

Immunoblot analysis. Whole-cell extracts were prepared in urea buffer (6 M urea, 100 mM sodium dihydrophosphate, 10 mM Tris pH 8). SDS-PAGE was performed using TGX gradient gels (Bio-Rad) and transferred onto PVDF (Millipore) using TransBlot SD semi-dry transfer apparatus (Bio-Rad) as per manufacturer's guidelines. The blots were probed with following antibodies: p21, p53, and PUMA (Santa Cruz), HIF-1a (RandD Systems), LC3 (Novus), PKM2 (Cell Signaling), p62 and tubulin (Sigma). Blot images were captured on ImageQuant LAS 4000 digital imaging system (GE Healthcare, Piscataway, NJ).

Iron stain. Cells were seeded on 22 mm collagen-coated coverslips (BD) at the density of 3.5 x 10^5 per well of a 6-well plate. Next day, they were subject to treatment with vehicle (0.1% ethanol) or 20 uM MEN for 2 hours. Cells were fixed and stained simultaneously in 4% formaldehyde and Perls' solution (1% w/v K4Fe(CN)6 in 1% HCl) for 30 min at room temperature. After rinsing in PBS, cells were incubated with 0.75 mg/ml diaminobenzidine (DAB), 0.07% H2O2 in PBS for 30–60 min. The reaction was stopped by rinsing in PBS. Negative controls were prepared with the samples from control and MEN-treated cells subjected to the DAB exposure without the Perls' solution preincubation.

Measurement of lactate production. Lactate production was measured using a commercially available fluorescence-based assay kit (Cayman Chemical). Fresh media supplemented with 2% FBS was added to subconfluent cells grown in 6-well plates (Corning), and aliquots of media from each well were assessed for the amount of lactate present under indicated treatment conditions. The relative amounts of lactate were normalized to the mean number of cells of three independent cell counts of each sample.

HPLC analysis of total nucleotides. Following treatment, medium was aspirated and the cells washed three times with ice-cold PBS. Extraction was done by scraping the cells in 180 µl ice-cold acetonitrile followed by 420 µl cold water. The soluble and precipitated fractions were centrifuged at 16000 g for 10 min at -20° C. The supernatant fraction, kept on ice, was then gassed with N2 for 30 min to evaporate acetonitrile. The pellet was solubilized with 1 N NaOH and the protein content analyzed with Coomassie Blue assay (Pierce Chemical, Rockford, IL). The column used was a 4 µm Nova-Pack C18 cartridge (100 mm by 8 mm ID), equipped with a radial compression chamber (Waters, Millford, MA). The buffer consisted of 20% acetonitrile, 10 mM ammonium phosphate and 2 mM PIC-A ion pairing reagent (Waters) and was run isocratically at 2 ml/min (10). Samples were diluted in half and the injection volume was 100 µl. A HP Chemstation model 1100 was used (Hewlett-Packard, Wilmington, DE), and the UV detector set at 254 nm. HPLC grade nucleotide standards were used to calibrate the signals. They were run daily because the retention of the column varied with time. Internal standards were added to the samples to test recovery. It exceeded 90% for all nucleotides.

Reagents. Buparvaquone was purchased from AK Scientific, DMOG from Cayman Chemical Company, etoposide from Tocris, menadione and hydrogen peroxide from ACROS Organics, tramafenib and vemurafenib from Selleck Chemical. All other chemical compounds were purchased from Sigma.

siRNA knockdown. Gene knockdown was done using PepMute (SignaGen) with custom-made dsiRNA (IDT) based on published sequences. Briefly, cells were seeded into 6-well plates at a density of 3 x 10^5 cells per well. siRNA complexes were prepared at 20 nM siRNA in according to the manufacturer's instructions.

shRNA-mediated silencing. The lentiviral shRNA expression plasmids were from Sigma. The shRNAs targeting HIF-1a is TRCN0000010819 and ARNT is TRCN0000356097. The control shRNA is the pLKO.1 - TRC control (Addgene, plasmid 10879). The production of viral particles and transduction of target cells was conducted as described on http://www.broad.mit.edu/genome_bio/trc/publicProtocols.html.

Site-directed mutagenesis. PCR amplification of HIF1a-containing pcDNA3.1 plasmid with primers incorporating mutations coding for HIF1a 213Q was conducted using Phusion High-Fidelity PCR enzyme (Thermo-Fisher), following manufacturer's protocol.

Live cell imaging. To determine mitochondrial membrane potential, cells were incubated with 5 nM Tetramethylrhodamine methyl ester (Life Technologies). Time lapse images were obtained with a custom spinning-disk microscope built around a CSU-10 Confocal head (Yokogawa), a 897 Xion EMCCD camera (Andor), a 4-line monolithic laser launch (Agilent) and a TiE inverted microscope stand (Nikon Instruments) equipped with a stage top incubator that regulates CO2 and temperature.

Statistical Analysis. The DHE fluorescence plots show F/F0 traces from individual cells (thin, grey traces) as well as the average F/F0 signal (thick, red trace). The average DHE F/F0 traces are mean ±SEM.
